# Perspective: Role of Micronutrients and Omega-3 Long-Chain Polyunsaturated Fatty Acids for Immune Outcomes of Relevance to Infections in Older Adults—A Narrative Review and Call for Action

**DOI:** 10.1093/advances/nmac058

**Published:** 2022-05-19

**Authors:** Manfred Eggersdorfer, Mette M Berger, Philip C Calder, Adrian F Gombart, Emily Ho, Alessandro Laviano, Simin N Meydani

**Affiliations:** Department of Internal Medicine, University Medical Center Groningen, Groningen, The Netherlands; Lausanne University Hospital, Lausanne, Switzerland; Faculty of Medicine, University of Southampton and NIHR Southampton Biomedical Research Centre, University Hospital Southampton NHS Foundation Trust, Southampton, United Kingdom; Department of Biochemistry and Biophysics, Linus Pauling Institute, Oregon State University, Corvallis, OR, USA; College of Public Health and Human Sciences, Linus Pauling Institute, Oregon State University, Corvallis, OR, USA; Department of Translational and Precision Medicine, Sapienza University, Rome, Italy; Nutritional Immunology Laboratory, Jean Mayer USDA Human Nutrition Research Center on Aging at Tufts University, Boston, MA, USA

**Keywords:** older adults, vitamin, trace element, docosahexaenoic acid, eicosapentaenoic acid, viral infection, immunosenescence, inflammaging, influenza, COVID-19

## Abstract

The immune system is weakened by advancing age, often referred to as immunosenescence, increasing the vulnerability to, and frequently the severity of, infectious diseases in older people. This has become very apparent in the current coronavirus disease 2019 (COVID-19) pandemic for which older people are at higher risk of severe outcomes, even those who are fully vaccinated. Aging affects both the innate and adaptive immune systems and is characterized by an imbalanced inflammatory response. Increasing evidence shows that optimal status of nutrients such as vitamins C, D, and E and selenium and zinc as well as the omega-3 (n–3) fatty acids DHA and EPA can help compensate for these age-related changes. While inadequate intakes of these nutrients are widespread in the general population, this is often more pronounced in older people. Maintaining adequate intakes is a challenge for them due to a range of factors such as physical, physiological, and cognitive changes; altered absorption; and the presence of noncommunicable diseases. While nutritional requirements are ideally covered by a balanced diet, this can be difficult to achieve, particularly for older people. Fortified foods and nutritional complements are effective in achieving adequate micronutrient intakes and should be considered as a safe and cost-effective means for older people to improve their nutritional status and hence support their defense against infections. Complementing the diet with a combination of micronutrients, particularly those playing a key role in the immune system such as vitamins C, D, and E and selenium and zinc as well as DHA and EPA, is recommended for older people. Optimal nutrition to support the immune system in older people will remain essential, particularly in the face of the current COVID-19 pandemic and, thus, developing strategies to ensure adequate nutrition for the growing number of older adults will be an important and cost-effective investment in the future.

## Teaser

**Statement of Significance**: Prevention and treatment of age-associated decline of immunity contribute to successful aging. Key nutrients have been proposed and suggested to play a role in supporting immunity in older adults. In contrast with published reviews in which nutrients are discussed separately, this Perspective summarizes the available literature on nutrients and immunity and proposes a novel integrative approach, in which recommended nutrients should be provided in combination to maximize their effects.

## Introduction

The current severe acute respiratory syndrome coronavirus 2 (SARS-CoV-2) coronavirus disease 2019 (COVID-19) pandemic has highlighted the vulnerability of older adults to infections, as they have a significantly increased risk for a severe as well as a fatal course of the disease (1, 2). Moreover, their response to vaccines is less pronounced, as shown in a study where one-third of the elderly had no detectable neutralizing antibodies after the second dose of the BNT162b2 COVID-19 vaccine (BioNTech and Pfizer) in contrast to only 2.2% of those aged <60 y (3). Similarly, a negative association between antibody titers and age was found despite a high (96%) overall response to the vaccine (4). Before the COVID-19 pandemic, seasonal influenza caused an estimated 3 to 5 million cases of severe illness globally every year, resulting in 290,000 to 650,000 deaths, and older adults were also at increased risk of severe outcomes for this viral infection (5).

Nutrition is essential for a well-functioning immune system in the general population (6) but may be particularly important for older people who exhibit a dysregulated immune response as well as nutritional insufficiency (7). Therefore, in this review we aim to explore the role of complementing the diet with specific nutrients that older people have low intakes of and that are particularly relevant for a well-functioning immune system. We will focus on specific nutrients [vitamins C, D, and E; selenium; zinc; and omega-3 (n–3) long-chain PUFAs DHA and EPA], important for maintaining an efficient immune defense against bacterial and viral infections at an advanced age.

## The Aging Immune System

The immune system is composed of innate and adaptive responses and most of these are affected by advancing age (8). The term “immunosenescence” refers to the most marked changes that occur with aging in the adaptive immune system, responsible for increased susceptibility to new infections. “Inflammaging” is the long-term result of the chronic physiological stimulation of the innate immune system, which can become dysregulated during aging, the by-product of the degeneracy of a few receptors that can sense a variety of non-self, self, and quasi-self damage signals (9). Immunosenescence is driven by a range of factors, such as reduced immune cell output from the bone marrow and thymus, cell senescence, mitochondrial dysfunction, and oxidative stress (1). Physical barriers, such as skin, mucus, and gut epithelium, are components of the innate immune system that help prevent pathogen entry. As regeneration capacity of cells and tissues decreases with age, barrier function weakens and hence is less efficient at preventing pathogen entry into the body (10).

Many important effector functions of neutrophils and monocytes/macrophages, including phagocytosis, chemotaxis, and cytotoxicity, are altered, even in apparently healthy aging (8). A variety of phagocytes and killer cells, cytokines, and proteins quickly recognize and neutralize or destroy invading pathogens, through a coordinated effort between cell-mediated and inflammatory processes, and then resolve the inflammation and repair the damage caused by these processes (11). While the inflammatory response can still be initiated at an older age, its resolution is often impaired. A vicious circle has been described where immunosenescence and inflammaging [i.e., the long-term result of the chronic physiological stimulation of the innate immune system (9)] contribute to age-associated chronic disease, which, in turn, may lead to inflammatory burden and an impaired immune system. Of note, inflammaging is also influenced by other factors such as type of diet and probiotics, which are beyond the scope of this review. The oxidative burst is another important component of immune defense, where reactive oxygen species are produced and antioxidant mechanisms are required to prevent damage to host tissues (12).

**FIGURE 1:**
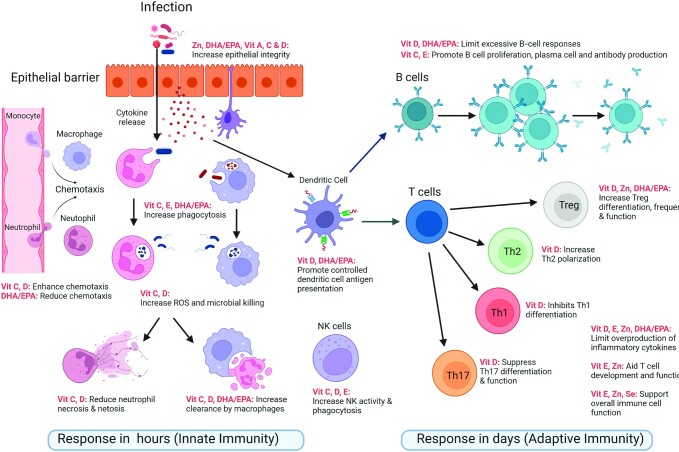
Temporal interaction between cells of the innate and adaptive immune systems and how their functions are affected by the vitamins A, C, D, and E; the trace elements selenium and zinc; and omega-3 PUFAs DHA and EPA. For further vitamin A data, see reference (99). Th, T-helper; Treg, T-regulatory; Vit, vitamin. (Created with BioRender.com.)

If the innate defenses cannot resolve an infection, the adaptive response takes over. The timely changes and micronutrient actions are summarized in **Figure 1**: subsets of T lymphocytes coordinate the overall adaptive response or kill infected cells, and B lymphocytes are activated to secrete antibodies specific to the infecting pathogen (11). In addition, the T and B lymphocytes are responsible for generating immunological memory, whereby a repeated infection will generate a fast and vigorous adaptive response.

Activation of the adaptive response by the innate immune system is frequently impaired with older age and the overall immune response is less efficient (8). For example, impaired cytokine regulation combined with decreased viral clearance increases the risk of symptom severity and fatality seen in older people (13). The thymus, a central lymphoid organ and the site of T-cell maturation, shrinks with advancing age, resulting in a gradual decline in both cellular and humoral immune responses (1). This is accompanied by a shift in the number of naive compared with memory T cells as well as changes in their signaling cascades (8). As a result, responses to new infections as well as previously encountered pathogens are less potent than in younger adults. Consequently, the risk of infection increases, and they tend to last longer, are more severe, and result in more complications (7). Moreover, as mentioned above, the efficacy of some vaccines decreases with aging: antibody response to the seasonal influenza vaccine, for example, was significantly lower (17–53% protection) in older people compared with younger adults (70–90% protection) (14). One could argue that many of these age-related changes are involved in the increased vulnerability of older people, particularly those with comorbidities, to viral infections as seen in the current COVID-19 pandemic (15). Vaccines are increasingly available globally to control SARS-CoV-2 infections, but their efficiency, and likely the protective period, appears to be decreased in older people. There is still no cure for the disease and new virus mutations will likely continue to emerge; thus, it is possible that current vaccines will not effectively combat these new strains. Therefore, supporting the immune system with optimal nutrition is an important public health measure in addition to other measures such as good hygiene practices and social distancing.

## Specific Challenges for Older Adults to Achieve Adequate Intakes

Ideally, optimal intakes of nutrients are achieved through the consumption of a well-balanced diet. However, this is a challenge in the general population, and older adults are even less likely to ingest the required amounts of energy and micronutrients, in part due to the so-called anorexia of aging (16). The loss of appetite and/or decreased food intake often observed in advanced age is a complex, multifactorial geriatric syndrome involving physical, physiological, cognitive, and social factors associated with the aging process as well as noncommunicable diseases (16). While the energy requirements of older adults tend to decrease compared with those for younger people, micronutrient needs remain similar or are even higher (17–20). Moreover, requirements for specific nutrients such as vitamin D and long-chain n–3 PUFAs are in a range unlikely to be met by the diet alone (21). Even in affluent countries, inadequate status or deficiencies in a range of micronutrients are observed in a significant proportion of the older population (22).

A recent publication of NHANES data from the United States shows that the use of micronutrient supplements can decrease significantly the proportion of adults who have intakes of micronutrients below the recommended levels: with supplement use, the percentage of the population with usual intakes below the Estimated Average Requirement (EAR) in older adults (≥71 y) for vitamin A, vitamin C, vitamin D, vitamin E, and zinc decreased from 37% to 23%, 44% to 25%, 96% to 44%, 92% to 52%, and 26% to 16%, respectively (23).

Decreased appetite, lack of energy, and depressive symptoms are normal physiological reactions to the release of proinflammatory cytokines, but these symptoms tend to be more pronounced in older adults (8). Moreover, infections frequently lead to decreased intestinal absorption, catabolic losses, and increased energy and nutrient requirements to fuel the immune response (24). This results in a vicious cycle where poor nutrition impairs the defense against infection, and infections lead to a further deterioration of nutritional state (24).

## Nutritional Support for Immunity in Older Adults

The importance of essential nutrients in maintaining a well-functioning immune system is well established and was reviewed recently elsewhere (6, 7). An adequate intake of micronutrients is consequently recommended for all individuals and, in particular, those at increased risk of a severe course of COVID-19 infection, including older adults (25). A recent publication recommends multivitamin and trace element supplements in combination with at least 200 mg vitamin C/d, 2000 IU vitamin D/d, 8–11 mg zinc/d, and 250 mg DHA + EPA/d to support a well-functioning immune system in the general population (6).

While an adequate supply of all nutrients is essential for an optimal immune function, this review will focus on the ability of complementation with vitamins C, D, and E and selenium and zinc as well as DHA and EPA to counter the negative effects of immunosenescence.

Other nutrients, such as the B-vitamins, copper, iron, and magnesium, are essential to sustain a strong immune system, but the available data in older adults are lacking to draw firm conclusions on their role in countering immunosenescence.

### Vitamin C

Vitamin C is a potent antioxidant and contributes to reduce the inflammatory processes. It plays a key role in immune defense as it is required by cells of the innate and the adaptive immune systems and helps protect the body from damage as a consequence of inflammatory responses (26). Vitamin C promotes barrier function; the function of neutrophils, monocytes, and macrophages; the activity of NK cells; the differentiation and function of T cells, especially cytotoxic T cells; and production of antibodies (26). Deficiency increases the susceptibility to infections such as pneumonia (26) and blood concentrations decrease during an acute infection (27). Moreover, a trend towards lower vitamin C concentrations in the plasma, platelets, and immune cells was reported in the most severely ill older patients admitted to the hospital with acute respiratory infection (27). Serum concentrations were inversely associated with overall mortality, resulting in an HR of 0.54 (95% CI: 0.34, 0.84) between the highest quintile (>66 μmol/L) and the lowest quintile (<17 μmol/L) in older adults in the United Kingdom (28). Randomized controlled trials supplementing vitamin C in older people to improve their ability to deal with infections are relatively scarce (**Table 1**). Trials vary significantly in factors such as participant characteristics and the dose, duration, and outcomes. In some cases, vitamin C was given in combination with other micronutrients (29–31), complicating the interpretation of the data. In the trial by Thomas et al. (31), a wide range of ages was included, making conclusions about the effects in older people difficult. Despite insufficient evidence from human studies, the combination of these data with the preclinical and epidemiological evidence on the increased requirements and the critical role of vitamin C in the immune system suggests that assuring adequate intakes of vitamin C will be prudent for older people.

**TABLE 1:** RCTs of supplementation with micronutrients in older adults on immune response^1^

Author, year (reference)	Micronutrient	Participants	Intervention and control group	Baseline serum concentration, mg/dL	Endpoint serum concentration, mg/dL	Immune-related results
Hunt et al., 1994 (27)	Vitamin C	Older patients (66–93 y); acute respiratory infections	0 (*n* = 29) or 200 mg/d (*n* = 28); 4 wk	0.41 ± 0.40^2^	PG: 0.43 ± 0.35^2^IG: 1.67 ± 0.57^2,*^	Increased plasma and white blood cell vitamin C concentration even during infection; better recovery, particularly in those most severely ill on admission
Sasazuki et al., 2006 (100)	Vitamin C	Patients; chronic atrophic gastritis (40–69 y; mean 57 y)	50 (*n* = 120) or 500 mg/d (*n* = 124); 5 y	LD: 1.38 ± 0.03^2^HD: 1.35 ± 0.03^2^	NA	Reduced risk (RR: 0.34; 95% CI: 012, 0.97) of suffering from a common cold ≥3 times during the survey period; no effect on severity and duration of common cold
Thomas et al., 2021 (31)	Vitamin C	Patients; SARS-CoV-2 infection (≥18 y)	0 (*n* = 108) or 8000 mg/d with or without 50 mg zinc (*n* = 106); 10 d	NA	NA	No effect on time to reach 50% reduction in symptoms, composite 4-symptom score, hospitalization, or death
Aloia and Li-Ng, 2007 (101)	Vitamin D	Postmenopausal African-American women (50–75 y)	0 (*n* = 104) or 800 IU/d (2 y) + 2000 IU/d (1 y) (+ Ca for 3 y) (*n* = 104)	NA	NA	Reduced reported cold and influenza symptoms (*P* < 0.002)
Bischoff-Ferrari et al., 2010 (42)	Vitamin D	Older people; recent hip fracture (≥65 y)	800 (*n* = 89) or 2000 IU/d + Ca; 3 mo (*n* = 100)	HD: 13.2 ± 8.1^2^LD: 12.3 ± 7.7^2^	HD: 44.7 ± 10.4^2^LD: 35.4 ± 10.1^2^^,*^	Decreased number of participants with hospital readmission due to infection (–39%; 95% CI: -62%, -1%)
Li-Ng et al., 2009 (43)	Vitamin D	Adults (18–80 y)	0 (*n* = 70) or 2000 IU/d; 3 mo (*n* = 78)	IG: 25.7 ± 11.48^2^PG: 25.2 ± 10.32^2^	IG: 35.5 ± 9.3^2^PG: NAΔ IG: 9.6 (95% CI: 8.7, 12.3)^3^Δ PG: –0.84 (95% CI: –2.8, 1.1)^3^^, *^	No effect on duration or severity of upper respiratory infection symptoms
De la Fuente et al., 2008 (102)	Vitamin E	Healthy older adults (70.4 ± 5.1 y)	200 mg/d (*n* = 33) or CG (29.7 ± 4.9 y) (*n* = 30); 3 mo	NA	NA	Normalization of a range of immune parameters (lymphocytes, neutrophils, etc.)
Pallast et al., 1999 (103)	Vitamin E	Healthy older adults (65–80 y)	0 (*n* = 50), 50 (*n* = 54), or 100 mg/d (*n* = 53); 6 mo	PG: 1.27 ± 0.30^2^LD: 1.24 ± 0.30^2^HD: 1.34 ± 0.26^2^^v^	ΔPG: 0.02 ± 0.16^2^ΔLD: 0.44 ± 0.22^2^^, *^ΔHD: 0.68 ± 0.32^2^^, *^	Trend to increased delayed-type hypersensitivity and IL-6 production with increasing dose of vitamin E
Meydani et al., 1990 (104)	Vitamin E	Healthy older adults (≥60 y)	0 (*n* = 14) or 800 mg/d (*n* = 18); 30 d	PG: 1.13 ± 0.07^2^IG: 1.10 ± 0.06^2^	PG: 1.03 ± 0.06^2^IG: 3.05 ± 0.27^2^^,*^	Increased peripheral blood mononuclear cells, delayed-type hypersensitivity skin test, mitogen-stimulated lymphocyte proliferation and other immune markers
Meydani et al., 1997 (47)	Vitamin E	Free-living healthy older adults (≥65 y)	0 (*n* = 19), 60 (*n* = 20), 200 (*n* = 20), 800 mg/d (*n* = 19); 235 d	PG: 1.06 ± 0.20^2^LD: 1.17 ± 0.26^2^ID: 1.10 ± 0.25^2^HD: 1.11 ± 0.27^2^	PG: 1.00 ± 0.09^2^LD: 1.65 ± 0.23^2^^,*^ID: 2.20 ± 0.59^2^^,*^HD: 3.08 ± 1.14^2^^,*^	Increased a range of markers of adaptive immunity, including delayed type hypersensitivity skin test and response to vaccination, plateauing at 200 mg/d
Graat et al., 2002 (48)	Vitamin E	Noninstitutionalized older adults (≥60 y)	0 (*n* = 153) or 200 mg/d (*n* = 164); 15 mo	PG: 1.25 ± 0.27^2^IG: 1.23 ± 0.27^2^	NA	No effect on incidence of acute respiratory tract infections; severity of infection was greater in the vitamin E group
Meydani et al., 2004 (49)	Vitamin E	Nursing home residents (≥65 y)	One-half of the RDA (*n* = 220) or 200 IU/d (*n* = 231); 1 y	PG: 1.15 ± 0.43^2^IG: 1.14 ± 0.39^2^	PG: 1.21 ± 0.41^2^IG: 2.12 ± 0.69^2^^,*^	No effect on incidence or number of days with infection for all, upper, or lower respiratory infections; fewer vitamin E–supplemented subjects acquired 1 or more respiratory infections or upper respiratory infections, common colds and shorter duration of colds
Hemilä et al., 2016 (105)	Vitamin E	Male smokers (50–69 y)	0 (*n* = 1265) or 50 mg/d (*n* = 1286); 5–8 y	NA	NA	Decreased incidence of pneumonia (–69%; 95% CI: –83%, –43%)
Hemilä et al., 2006 (106)	Vitamin E	Male smokers (50–69 y)	50 mg/d (*n* = NA) or control group (*n* = NA); 5–8 y	NA	NA	Reduced risk of common cold (RR: 0.54; 95% CI: 0.37, 0.80) in those who smoked 5–14/d cigarettes; increased risk (RR: 1.58; 95% CI: 1.23, 2.01) in those smoking more
Bentley-Hewitt et al., 2014 (107)	Selenium	Adults (24–65 y)	200 μg selenium/d from enriched broccoli; crossover with normal broccoli (*n* = 18); 3 d	F: 1.3 (0.97–1.72)^4^M: 1.2 (0.95–1.54)^4^	N.S. change from baseline; actual values NA	Increased several markers of immune response
Ivory et al., 2017 (108)	Selenium	82 adults (50–64 y)	0, 50, 100, or 200 μg/d; 12 wk	NA	NA	Mixed results on cellular immune response to influenza vaccine
Duchateau et al., 1981 (109)	Zinc	Institutionalized healthy older adults (>70 y)	100 mg/d (*n* = 15) or control group (*n* = 15); 1 mo	NA	NA	Increased some (e.g., number of circulating T lymphocytes), but not all markers of immune function measured
Bogden et al., 1988 (110)	Zinc	Community-dwelling older adults (60–89 y)	0 (*n* = 36), 15 (*n* = 36) or 100 mg/d + vitamin-mineral capsule (*n* = 31); 3 mo	PG: 85.0 ± 9.8^2^LD: 83.7 ± 19.0^2^HD: 85.6 ± 13.1^2^	PG: 81.7 ± 13.1^2^LD: 85.6 ± 13.7^2^HD: 109.8 ± 22.9^2^^,*^	No effect on delayed type hypersensitivity skin test
Bogden et al., 1990 (111)	Zinc	Community-dwelling older adults (60–89 y)	0 (*n* = 22), 15 (*n* = 20) or 100 mg/d + vitamin-mineral capsule (*n* = 19); 1 y	PG: 87.0 ± 2.6^2^LD: 84.3 ± 3.3^2^HD: 85.6 ± 3.3^2^	PG: 88.9 ± 2.6^2^LD: 87.0 ± 17.0^2^HD: 109.8 ± 3.9^2^^,*^	No effect on immune cell numbers; NK cell activity was transiently increased at the higher zinc dose; progressive improvement in delayed type hypersensitivity skin test
Cossack, 1989 (112)	Zinc	Zinc-deficient older adults (60–89 y)	60 mg/ d (*n* = 8); 4.5 mo compared with baseline	75 ± 15^2^	115 ± 19^2^^,*^	Increase in erythrocytes, lymphocytes, neutrophils, and erythrocyte nucleoside phosphorylase
Boukaïba et al., 1993 (113)	Zinc	Institutionalized older adults (73–106 y)	0 or 20 mg/d crossover (*n* = 44); 8 wk	HBW: 79.8 ± 2.9^2^LBW: 70.0 ± 1.8^2^	PG HBW: 81.7 ± 4.4^2^PG LBW: 69.3 ± 2.1^2^IG HBW: 94.8 ± 5.9^2^IG LBW: 88.0 ± 2.0^2^	Increased serum thymulin activity
Prasad et al., 1993 (114)	Zinc	Zinc-deficient older adults (50–80 y)	30 mg/d (*n* = 13); 6 mo compared with baseline	105.85 ± 14.8^2^	140.75 ± 31.48^2^^,*^	Increased delayed type hypersensitivity skin test and normalization of various immune markers
Fortes et al., 1998 (115)	Zinc	Retirement home residents (≥65 y)	0 (*n* = 61) or 25 mg/d ± vitamin A (*n* = 57); 3 mo	NA	NA	Increased cell-mediated immune response
Provinciali et al., 1998 (116)	Zinc	Institutionalized older adults (64–100 y)	90 mg/d (*n* = 33) or CG (*n* = 31); 60 d	CG: 69.7 ± 18.8^2^IG: 66.1 ± 13.6^2^	CG: 5.8 ± 14.8^2,5^IG: 89.0 ± 26.5^2^^,*^	No effect on immune parameters measured
Mocchegiani et al., 1999 (117)	Zinc	Older adults; chronic obstructive bronchitis (63–75 y)	0 (*n* = 14) or 12 mg/d (*n* = 15); 1 mo	76.8 ± 4.3^1^	NA	Increased CD4+ count
Mocchegiani et al., 2003 (118)	Zinc	Healthy older adults (63–75 y)	0 (*n* = 23) or 12 mg/d (*n* = 24); 1 mo	NA	NA	Restored NK cell activity and decreased incidence of severe infections
Kahmann et al., 2006 (119)	Zinc	Healthy older adults (65–82 y)	10 mg/d (*n* = 19); 7 wk compared with baseline	72.6 ± 2.7^2^	79.9 ± 2.3^2^^,*^	Decreased number of activated T-helper cells, but no change in ratio of Th1:Th2 cells
Hodkinson et al., 2007 (120)	Zinc	Healthy older adults (55–70 y)	0 (*n* = 31), 15 (*n* = 28), or 30 mg/d (*n* = 34); 6 mo	PG: 86.3 ± 1.8^2^LD: 85.0 ± 2.0^2^HD: 84.3 ± 1.2^2^	PG: 81.1 ± 1.8^2^LD: 85.0 ± 3.1^2^HD: 93.5 ± 5.0^2^^,*^	Increased ratio of CD4 to CD8 T lymphocytes, but no effect on long-term immune status
Meydani et al., 2007 (62)	Zinc	Nursing home residents (≥65 y)	7 mg/d (*n* = 420); 12 mo	NA	NA	Lower incidence of pneumonia; fewer new antibiotic prescriptions; shorter duration of pneumonia; fewer days of antibiotic use
Prasad et al., 2007 (121)	Zinc	Healthy older adults (55–87 y)	0 (*n* = 25) or 45 mg/d (*n* = 24); 12 mo	NA	NA	Lower incidence of infections, ex vivo generation of TNF-α and lower plasma markers for oxidative stress
Barnett et al., 2016 (69)	Zinc	Nursing home residents (≥65 y)	0 (*n* = 16) or 30 mg/d (*n* = 14); 3 mo	PG: 66.3 ± 10.0^2^IG: 63.9 ± 9.7^2^	PG: 65.4 ± 8.8^2^IG: 73.2 ± 14.6^2^^, a^	Increased T-cell function
Thomas et al., 2021 (31)	Zinc	Patients; SARS-CoV-2 infection (≥18 y)	0 (*n* = 50) or 50 mg/d with or without 8,000 mg vitamin C (*n* = 116); 10 d	NA	NA	No effect on time to reach 50% reduction in symptoms, composite 4-symptom score, hospitalization, or death
Girodon et al., 1997 (29)	Multiple vitamins and trace elements	Nursing home residents (≥65 y)	0 (*n* = 20) or 120 mg/d vitamin C, 6 mg/d β-carotene, 15 mg/d vitamin E, 20 mg/d Zn, 100 μg/d Se (*n* = 21); 2 y	Improvement in the status for the supplemented nutrients		Decreased incidence of respiratory and urogenital infections (*P* <0.01); no effect on mortality
Girodon et al., 1999 (30)	Multiple vitamins and trace elements	Nursing home residents (≥65 y)	0 (*n* = 182) or 120 mg/d vitamin C, 6 mg/d β-carotene, 15 mg/d vitamin E, 20 mg/d Zn, 100 μg/d Se (*n* = 181); 2 y	Significant improvement in α-tocopherol, β-carotene, vitamin C, Zn, and Se status		Increased antibody response to influenza vaccine; higher number of serologically protected patients; no effect on the incidence of respiratory tract events
Lenhart et al., 2020 (123)	Multiple vitamins and trace elements	Healthy (asymptomatic) adults (18–65 y)	Placebo (*n* = 130) or multi-micronutrient supplement (129); 12 wk	NA	NA	Trend for reduced odds of upper respiratory infection (*P* = 0.14); lower odds of reporting symptoms for runny nose (OR: 0.53; *P* = 0.01) and cough (OR: 0.51; *P* = 0.04); no effect on severity
Schmoranzer et al., 2009 (124)	Multiple vitamins and trace elements	Residents of nursing homes (62–98 y)	Placebo (*n* = 42) or micronutrient supplement (*n* = 40); 3 mo	NA	NA	Increased number of various types of immune cells; no effect on specific antibody response to influenza vaccination

1 * *P* < 0.05. CG, control group; HBW, high body weight (BMI ≥24 kg/m^2^); HD, high-dose group; ID, intermediate-dose group; IG, intervention group; LBW, low body weight (BMI ≤21 kg/m^2^); LD, low-dose group; NA, not available; PG, placebo group; RCT, randomized controlled trial; SARS-CoV-2, severe acute respiratory syndrome coronavirus 2.

2 Mean ± SD.

3 Mean (95% CI).

4 Mean (range).

5 Reported in error as 5.8 ±14.8 μg dL.

A recent meta-analysis reported that vitamin C supplementation led to a significant reduction in the risk of pneumonia, particularly in individuals with low dietary intakes in a range of age groups (32). There is also evidence for an effect in the prevention and treatment of respiratory tract infections, such as the common cold, in the general population (33). A recent review concluded that, for individuals admitted to the hospital with community-acquired pneumonia, vitamin C may improve respiratory function in more severe cases, without any adverse effects (34). On the other hand, it should be noted that a recent study reported that treatment with high-dose zinc gluconate, ascorbic acid, or a combination of the 2 supplements did not significantly decrease the duration of COVID-19 symptoms compared with standard of care (31).

It has been proposed that persons with noncommunicable diseases may have higher requirements of vitamin C, given the oxidative potential of such conditions (35). Pooled data on the association between vitamin C intake and resulting serum concentrations showed that older adults (aged 60–96 y) achieve lower blood concentrations with a given vitamin C intake compared with younger individuals (aged 15–65 y) (36). This led the authors to conclude that older adults have higher vitamin C requirements. For healthy older people, intakes of at least 200 mg/d proposed as optimal for healthy individuals in general (6) are recommended for immune system support until more evidence is available for this population group.

### Vitamin D

Inadequate or deficient serum 25-hydroxyvitamin D [25(OH)D] concentrations are frequent in older persons, likely due to reduced exposure to sunlight and synthesis in the skin, increased adiposity, low appetite and food intake, as well as impaired vitamin D absorption in the gut. Moreover, the decreasing concentration of 7-dehydrocholesterol in the skin with aging results in a 50% reduction in pre-vitamin D synthesis by older people in response to UV light (37). With its hormone-like functions, vitamin D is a key nutrient for a range of functions for the innate as well as adaptive immune system and helps mitigate the negative effects of inflammation, as reviewed elsewhere (38). Vitamin D has complex regulatory effects on many cells of the immune system, as reviewed elsewhere (39). It promotes production of antimicrobial proteins such as cathelicidin, the differentiation of monocytes to macrophages, and macrophage phagocytosis. Vitamin D promotes antigen processing, but it can inhibit antigen presentation. It promotes the development of regulatory T cells and regulates the function of other T-cell types and of B cells. Deficiency is associated with a general dysregulation of the immune system and increasing data suggest antiviral properties as well as a role in protecting against infections, including respiratory illnesses. A meta-analysis in the general population reported that daily or weekly vitamin D administration reduced the incidence of acute respiratory tract infections among all participants, but particularly in those with low serum 25(OH)D concentrations (40). A more recent meta-analysis by the same group including additional data found that this effect was strongest at daily intakes of 400 to 1000 IU vitamin D and they did not observe significant protective effects in those individuals with the lowest serum 25(OH)D concentrations at baseline (41). However, many of the studies using doses <2000 IU were done in children, while the studies in adults tended to use higher doses. Mechanistic studies in individuals randomly assigned to different dosing regimens of vitamin D are needed to clarify the findings from these meta-analyses.

The data from the limited number of randomized controlled trials for vitamin D with and without calcium available in older people are summarized in Table 1. One of these trials reports that a large majority of participants (97.7%) had baseline serum 25(OH)D concentrations below 30 ng/mL, which was successfully corrected with 3 mo supplementation with 2000 IU/d (97%, >30 ng/mL) and to a lesser degree with 800 IU/d (81%, >30 ng/mL) (42). One of the 3 trials (Table 1) found no effect, but the authors argue that supplementation might have started too close to the beginning of the cold season, and that baseline concentrations were not sufficiently low to see an effect (43).

Based on the available evidence in the general population, a previous expert panel recommended 2000 IU/d for adults to optimize the immune response, particularly against viral infections (6). Given the increased susceptibility of older people to inadequate 25(OH)D status, an intake of 2000 IU vitamin D/d as a supplement is recommended. In line with this, mounting evidence indicates an increased susceptibility to COVID-19 as well as an increased risk for a complicated course of the disease in individuals with insufficient 25(OH)D concentrations (44), although the findings are confounded by the observation that those deficient in vitamin D were also older adults. A number of well-designed studies including randomized controlled clinical trials are currently underway to define the best practice for use of vitamin D supplementation in the context of COVID-19 (44). In the meantime, vitamin D supplementation for older people or other at-risk groups is recommended as a cost-effective, available tool, particularly when combined with other public health measures. Supporting this recommendation, Louca et al. (45) reported in women a modest but significant association between the use of probiotics, n–3 fatty acids, multivitamin or vitamin D supplements and a lower risk of testing positive for SARS-CoV-2.

### Vitamin E

Vitamin E impacts various components of the immune system, including phagocytosis, T-cell proliferation and differentiation, antibody production, and modulation of inflammatory responses (46). This is likely via its role in reducing oxidative damage to cell membranes and in correcting age-associated dysregulation of redox status and via its modulatory effect on specific properties of cell membranes (46). Even though the aging process appears to spare the absorption of vitamin E (18), available evidence suggests increased requirements for this nutrient in older people: while vitamin E supplementation was shown to improve a range of immune markers at varying levels (Table 1), a dose–response study showed that 200 mg/d evoked the greatest increase in delayed-type hypersensitivity response and the largest response to hepatitis vaccination compared with the placebo (47). While this intake is significantly above the current recommendations, it is still well below vitamin E upper level (1000 IU/d) (18) and can be regarded as safe (46). No adverse effects were observed (biochemical, disease incidence, mortality) in the study by Meydani et al. (47). However, one study reported that intervention with 200 mg vitamin E/d in noninstitutionalized older people increased the severity of infections (48). This was attributed to a pro-oxidative effect of vitamin E in the absence of sufficient antioxidants such as vitamin C and glutathione to recycle the oxidized vitamin E (48). In fact, in another study conducted in 647 older adults residing in nursing homes in the United States, supplementation with 200 mg vitamin E/d in conjunction with one-half of the RDA of essential vitamins and minerals (to ensure adequate intake of all other nutrients), significantly reduced all upper respiratory infections, in particular common cold, compared to the control group who received one-half of the RDA of all vitamins and minerals (49). This highlights the importance of determining the impact of optimal intakes of a particular nutrient on immune response while other nutrient requirements are met.

### Selenium

Selenium plays a role in a range of immune functions via selenoproteins primarily involved in antioxidant defense, cell signaling, and redox homeostasis (50). Selenium affects both the innate as well as the adaptive immune response via its role in, for example, the activation of B and T cells and hence improved response to vaccines and in mechanisms such as oxidative burst, cytokine production, and the regulation of inflammation (51). Administration was shown to promote immune responses in several preclinical studies, as indicated by a range of markers such as T-cell proliferation and activity of NK cells, and is most effective if selenium status is low (51). Selenium status inadequacy or even deficiency is common on the European continent (52), and in other parts of the world, due to low soil content; this may contribute to alterations of immunity.

The evidence for the effect of selenium on viral infections and the proposed mechanisms have recently been reviewed (53, 54), highlighting the important role that selenium plays concerning host-, but also pathogen-, related factors. Coxsackie virus B3, an infectious cofactor for Keshan disease first described in northeastern China, was found to become more virulent if the host is selenium deficient (55). Ensuring adequate selenium status might therefore not only support the host immune system but also affect the virus itself and consequently the severity of the disease (56). It was suggested as a preventive agent for SARS-CoV-2 (57).

Evidence, albeit limited and somewhat inconsistent, indicates a beneficial effect of selenium complements in older persons (Table 1). The Institute of Medicine set the Tolerable Upper Intake Level at 400 μg selenium/d (18) and toxicity seems dependent on the form of selenium used and host status (57). Still, as selenium has a relatively narrow safety range, additional intake via supplements in the range of 50 to 100 μg selenium/d to increase concentrations to normal for those with low selenium status is recommended.

### Zinc

With its presence in a wide range of enzymes and transcription factors and its role in the regulation of intracellular signaling pathways, zinc affects both the innate and adaptive immune systems. Zinc promotes barrier integrity. It supports monocyte and macrophage phagocytosis and NK cell activity and promotes the activity of T-helper 1 (Th1) lymphocytes, the proliferation of cytotoxic T cells, the development of regulatory T cells, and the production of antibodies (58). Zinc also has specific antiviral actions (59). Deficiency is thought to result in immune dysfunction, including thymic atrophy, lymphopenia, and impaired adaptive immunity (60). Zinc is crucial in the defense against viruses as it can inhibit the entry of certain viruses into the host cell through stabilization of the cell membrane, and it can interfere with their ability to replicate (61).

A significant percentage of older adults (30% and 22% in nursing home and independently living older adults, respectively) exhibit low serum zinc concentrations, and those with low serum zinc concentrations have higher pneumonia incidence compared with older adults with adequate serum zinc concentrations (62). Several randomized controlled trials assessed the effect of zinc supplementation on a range of markers of the immune system in older people (Table 1). Even though there are some inconsistencies, these studies support the important role zinc plays in the immune system in general, but also for older people. Importantly, zinc deficiency also manifests as an imbalanced inflammatory response and increased oxidative stress (63), further contributing to the negative effects of inflammaging and associated morbidities.

A review of available studies using a range of biomarkers such as serum zinc concentrations, or zinc concentrations in specific immune cells, showed a decrease in zinc status with age (64–66). This age-related decline is thought to be more dependent on physio-pathological changes occurring with aging rather than nutritional intake (67): the data even suggest that interventions with zinc supplements are required for health and longevity at an advanced age (68). Given the high risk for, and the severe immunological impact of, zinc deficiency in older adults, complements in the range of 8 to 11 mg zinc/d are recommended. Higher levels are needed for older adults with low serum zinc concentrations (69).

### DHA and EPA

The importance of DHA and EPA to support human health in general is well known and their role in the immune system is increasingly evident (70). In the body, DHA and EPA are incorporated into the phospholipid bilayer of cell membranes, where they affect different aspects of cell function (71). They play an important role in cell signaling and neurotransmission, cell division, gene expression, and lipid mediator production (72). Moreover, they are thought to enhance skeletal muscle anabolism, and play an important role in maintenance of muscle mass and function (73), which is key for healthy aging. Incorporation of EPA and DHA into cell membranes is typically at the expense of n–6 fatty acids, including arachidonic acid (20:4n−6). This is very important from the perspective of supporting immune function and controlling inflammation. Some eicosanoid mediators produced from arachidonic acid such as prostaglandin (PG) E2 have immunosuppressive effects, decreasing the function of T and B cells, while PGE2, PGD2, and several of the 4-series leukotrienes are involved in the inflammatory response (74). EPA and DHA act to decrease the production of these n–6 fatty acid–derived mediators and this is one mechanism whereby they can support adaptive immune responses and control adverse inflammatory responses (75). EPA and DHA have other actions in inflammatory pathways—for example, inhibiting activation of the NOD-, LRR- and pyrin domain-containing protein 3 inflammasome and of the NF-κB pathway (75). Such effects are important in regulating antiviral immune responses. An important recent discovery is that both DHA and EPA are substrates for the synthesis of highly active lipid mediators involved in regulating inflammatory processes and responses (76). Termed specialized pro-resolving mediators, they support the resolution of inflammatory processes by enhancing phagocytosis and decreasing production of inflammatory cytokines, chemokines, adhesion molecules, proteases, and enzymes (77). As a result, they encourage healing, which may consequently be hampered in case of nutritional deficiencies of DHA and EPA (78). The mitigation of adverse effects of inflammation through adequate intakes of DHA and EPA might therefore be particularly pronounced in older people (79).

In addition, increasing evidence indicates direct antimicrobial action of bioactive lipids such as DHA and EPA by inducing leakage in the cell membranes of pathogens (80). DHA and EPA increase the phagocytic capacity of macrophages and other cells to remove debris from the site of infection and injury and enhance microbial clearance (81).

Endogenous synthesis of EPA and DHA from α-linolenic acid (18:3n−3) is limited in most people and is influenced by a range of factors such as age, sex, genetics, and disease (82). Conversion is also impaired in conditions such as insulin resistance (83). This further emphasizes the importance of adequate intake of preformed DHA and EPA in older people who frequently suffer from insulin resistance or other features that limit endogenous synthesis.

It is now generally accepted that an intake of 250 to 500 mg/d EPA and DHA is required for optimal nutrition and supplements of up to 3000 to 5000 mg/d are regarded as safe (6, 84–87). Global mapping indicates low or even very low blood concentrations of n–3 PUFAs (i.e., DHA and EPA) in a large proportion of people for whom data were available (88). This will likely apply to older people to a similar or even higher degree given their difficulties meeting nutritional recommendations in general. Although few randomized controlled trials have assessed the effect of DHA and EPA on the immune system in older people, a complementary intake of up to 500 mg/d of the n–3 PUFAs EPA + DHA is recommended for older people based on the available evidence for their role in the immune system and emerging evidence (45).

## Conclusions and Call for Action

Older people are at increased risk of infections, and of infections being more severe, and they exhibit reduced responses to vaccines than younger adults (8). Suboptimal nutritional intakes and malnutrition do contribute to the age-related deterioration of the immune system and may contribute to further deterioration, particularly during a hospital stay or prolonged bedrest due to an illness (7). Western diets are high in saturated and n–6 fatty acids and refined carbohydrates and simple sugars, while being low in fiber and also often in micronutrients and other important nutrients like EPA and DHA (89). The imbalanced consumption of these nutrients can result in adverse impacts on metabolic processes (90) as well as on immune function and inflammatory processes (79, 91, 92). Thus, when considering the impact of single or multiple micronutrients and bioactive n–3 fatty acids on immunity, inflammation, and antiviral defenses, it is important to recognize that background diet is an important variable; a strongly “Western” style diet may add a layer of harm to the healthy aging process that already sees declines in appetite; in intake, absorption, and metabolism of key nutrients; and in immune defenses. Single-micronutrient supplementation as a treatment of active infection has not produced convincing results. An adequate nutrition (energy and proteins) can exert preventive effects by supporting the immune system and improving its ability to defend against infections. While the results from human studies are somewhat inconsistent and limited, the totality of evidence supports the role of complementary micronutrients and n–3 PUFAs. Only a few randomized controlled trials have tested the effect of multiple micronutrient supplements on the immune system in older people (Table 1). However, given the interdependence between nutrients for optimal function, it is prudent to ensure adequate intakes for all of them to eliminate intake gaps.

Many vitamins and trace minerals have recognized immunomodulatory actions and a variety of observational studies report that adequate micronutrient status or micronutrient supplementation is associated with enhanced vaccine responses, including to COVID-19 vaccination. A systematic review and meta-analysis of 9 clinical studies found lower seroprotection rates in people who were vitamin D deficient compared with those who were adequate, when vaccinated with H3N2 and B strains of seasonal influenza (93). A recent meta-analysis by Dissanayake et al. (94) demonstrates that, in people with vitamin D deficiency/insufficiency, the OR of developing COVID-19 is 1.46 (*P* < 0.0001), for developing severe disease is 1.90, and for death is 2.07. Holt et al. (95) studied the different risk factors for developing COVID-19 in the population-based longitudinal study (COVIDENCE UK) and identified that next to other risk factors, vitamin A, vitamin D, zinc, selenium, fish oil, and probiotic supplement users had a lower risk of COVID-19 infection. Jolliffe et al. (96) also report an independent association between vitamin D supplement use and enhanced humoral responses to COVID-19 vaccination. For some of these nutrients, requirements in older people are higher than in the general population due to reduced nutrient absorption and utilization, and to differing physiological status (e.g., inflammaging, immunosenescence). This is reflected in the recommendations for nutrient supplements to support the immune system in healthy older people (**Table 2**). Provision of these nutrients through supplements is safe, as they are well within the recommended upper safety limits set by expert authorities. For the needs of sick older patients who require repletion of micronutrients and n–3 PUFAs, the specific ESPEN guidelines should be consulted (97).

**TABLE 2:** Principal nutrients supporting the immune system in the general population

			DRI^2^	
Nutrient	Rationale	Suggested intake^1^	Recommended Dietary Allowances	Tolerable upper levels of intakes
Vitamins and trace elements	These micronutrients play important roles in supporting the cells and tissues of the immune system. Deficiencies or suboptimal status negatively affect immune function and can decrease resistance to infections	Daily multivitamin and trace element complements that supply the micronutrient requirements (e.g., DRI) are recommended in addition to the consumption of a balanced diet	Varies according to nutrient	
Vitamin C	Doses of ≥200 mg/d provide saturating concentrations in the blood, and support reduction in the risk, severity, and duration of upper and lower respiratory tract infections	Daily intake of at least 200 mg/d for healthy older adults	Men: 90 mg/dWomen: 75 mg/d	Men: 2000 mg/dWomen: 2000 mg/d
Vitamin D	Daily supplementation of vitamin D reduces the risk of acute respiratory tract infections. Dietary intake and endogenous synthesis is low in most older people	Intake of 2000 IU/d (50 μg/d)	Men: 20 μg/dWomen: 20 μg/d	Men: 100 μg/dWomen: 100 μg/d
Vitamin E	Emerging evidence indicates that current recommendations are too low to compensate for age-related losses and to counter imbalances in the immune system resulting in hyperinflammation in older people	A complementary intake of 200 IU/d^3^	Men: 15 mg/dWomen: 15 mg/d	Men: 1000 mg/dWomen: 1000 mg/d
Selenium	Selenium is important in the regulation of inflammation by preventing excessive and chronic inflammation and is essential for a well-functioning immune system	An intake from supplements in the range of 50 to 100 μg/d of selenium for those with low selenium status	Men: 55 μg/dWomen: 55 μg/d	Men: 400 μg/dWomen: 400 μg/d
Zinc	Marginal zinc deficiency can impact immunity. Those deficient in zinc are prone to increased diarrheal and respiratory morbidity	Daily intake in the range of 30 mg/d in those with low zinc status; a higher level maybe needed for older adults with very low serum zinc concentrations	Men: 11 mg/dWomen: 8 mg/d	Men: 40 mg/dWomen: 40 mg/d
DHA + EPA	DHA and EPA support an effective immune system, including by helping to resolve inflammation	Daily intake of up to 500 mg/d of EPA + DHA	NA	NA

1 Data from reference (6). NA, not available.

2 For those aged >70 years based on values from the Institute of Medicine (17, 18, 20).

3 One IU = 0.67 mg for *d-*ɑ-tocopherol (natural), 1 IU = 0.9 mg for *dl-*ɑ-tocopherol (synthetic).

More focus on nutritional support of the immune system is needed and public health officials are encouraged to advocate for nutritional strategies in supporting immunity in older adults. Health care budgets are strained in general and even more so during the current COVID-19 pandemic. Nutritional management, particularly of those at increased risk of nutritional inadequacies due to advanced age or pathologies such as cancer, should become or remain an integral part of longer-term public health programs. This applies to communities, hospitals, and nursing homes as it provides a promising, cost-efficient way to improve health outcomes.

To support immune responses in older adults, they should receive a yearly assessment of their individual nutrient status to tailor personalized interventions. For screening, validated public health nutrition tools such as the Mini-Nutritional Assessment Short Form should be used. These should be complemented with the assessment of micronutrient status in biological samples (e.g., blood or urine, as appropriate) to identify specific, likely marginal, deficiency. Optimal nutrition to support the immune system, particularly at an advanced age, will remain essential even after the current COVID-19 pandemic (98). Areas for future research should address the further elucidation of the role of micronutrient deficiencies and supplementation to vaccination efficacy. Hence, developing strategies to ensure optimal nutrition for the growing number of older adults to increase their health-span, and reduce health care costs associated with their care, will be an important and cost-effective investment in the future of our societies.

## Notes

The authors report no external funding received for this review. Funding for the vitamin E dose response and infection studies mentioned in this review was provided by USDA/Agricultural Research Service, NIH (to SNM). Hoffman La Roche provided the supplement for those studies but had no involvement in their design, execution, or publication.

Author disclosures: ME is president of the Gesellschaft für Angewandte Vitaminforschung (Germany), is a member of the scientific boards of PM International, and consults for companies in the nutrition and supplement field. MMB receives honoraria for lectures from Abbott, Baxter, B. Braun, DSM, Fresenius Kabi, Nestlé, and Nutricia, and has research funding from ESPEN, Fondation Nutrition 2000, and Swiss Foundation for Research. PCC has research funding from Bayer Consumer Care; acts as an advisor/consultant to BASF AS, DSM, Cargill, Smartfish, Nutrileads, Bayer Consumer Care, and GSK Consumer Healthcare; has received reimbursement for travel and/or speaking from Danone/Nutricia, Fresenius Kabi, Baxter, GSK Consumer Healthcare, Abbott, Smartfish, Biogredia, and the California Walnut Commission; and is past-President and member of the Board of Directors of the European Branch of the International Life Sciences Institute. AFG has received research funding from Bayer Consumer Care and has acted as an advisor/consultant for and has received reimbursement for travel and/or speaking from Bayer Consumer Care, GSK, DSM, Kellogg's, and The Coca-Cola Company. EH has received funding from Bayer Consumer Care and acts as an advisor to Oldways Whole Grains Co, and Pharmavite. AL has received research funding from Fresenius Kabi; acts as an advisor/consultant to Abbott, Baxter, B. Braun, DSM, Nestlé Health Science, Nutricia, and Smartfish; and has received honoraria for independent lectures by Abbott, Baxter, B. Braun, and Nutricia. SNM currently has research support from USDA, NIH, and the Gates Foundation and acts as an advisor to Nestle Research and Development, Danone Essential Dairy and plant-based scientific advisory board, Cargill Healthy Aging Group, Pharmavit, Arthritis Foundation, and the Barilla Health and Wellness Scientific Advisory Board.

Perspective articles allow authors to take a position on a topic of current major importance or controversy in the field of nutrition. As such, these articles could include statements based on author opinions or point of view. Opinions expressed in Perspective articles are those of the author and are not attributable to the funder(s) or the sponsor(s) or the publisher, Editor, or Editorial Board of *Advances in Nutrition*. Individuals with different positions on the topic of a Perspective are invited to submit their comments in the form of a Perspectives article or in a Letter to the Editor.

Abbreviations used: COVID-19, coronavirus disease 2019; PG, prostaglandin; SARS-CoV-2, severe acute respiratory syndrome coronavirus 2; 25(OH)D, 25-hydroxyvitamin D.
